# Fecal calprotectin levels in patients with non-celiac wheat sensitivity: a proof of concept

**DOI:** 10.1007/s11739-024-03595-7

**Published:** 2024-04-12

**Authors:** Aurelio Seidita, Alessandra Giuliano, Maurizio Soresi, Marta Chiavetta, Emilio Nardi, Giuseppe Mogavero, Giulio Giannone, Antonio Carroccio, Pasquale Mansueto

**Affiliations:** 1 Unit of Internal Medicine, “V. Cervello” Hospital, Ospedali Riuniti “Villa Sofia-Cervello”, Via Trabucco, 180, 90146 Palermo, Italy; 2 Department of Health Promotion Sciences, Maternal and Infant Care, Internal Medicine and Medical Specialties (PROMISE), University of Palermo, Palermo, Italy; 3 Institute for Biomedical Research and Innovation (IRIB), National Research Council (CNR), Palermo, Italy; 4 Unit of Internal Medicine, Department of Health Promotion Sciences, Maternal and Infant Care, Internal Medicine and Medical Specialties (PROMISE), University of Palermo, Palermo, Italy; 5 Unit of Internal Medicine II, Department of Health Promotion Sciences, Maternal and Infant Care, Internal Medicine and Medical Specialties (PROMISE), University of Palermo, Palermo, Italy; 6 Unit of Gastroenterology, “V. Cervello” Hospital, Ospedali Riuniti “Villa Sofia-Cervello”, Palermo, Italy; 7 Pathology Unit, Department of Health Promotion Sciences, Maternal and Infant Care, Internal Medicine and Medical Specialties (PROMISE), University of Palermo, Palermo, Italy

**Keywords:** Non-celiac wheat sensitivity, Diagnostic biomarker, Fecal calprotectin, Innate immune response, Intestinal inflammatory response, Gluten-free diet

## Abstract

Some data suggest the existence of intestinal inflammation in patients with non-celiac wheat sensitivity (NCWS). We aimed to verify whether fecal calprotectin (FCP), a marker of intestinal inflammation, could be used to confirm this inflammatory status and to test its diagnostic performance in differentiating NCWS from irritable bowel syndrome/functional dyspepsia (IBS/FD). We conducted a multicenter study, comparing NCWS patients, diagnosed by a double-blind placebo-controlled wheat challenge, with IBS/FD subjects. In the retrospective phase, FCP values were analyzed to define the prevalence of its positivity and its role as a NCWS diagnostic biomarker. In the prospective phase we tested the effects of a strict 6-month wheat-free diet (WFD) on FCP values. 31.3% (*n* = 63/201) of NCWS patients had above normal FCP values (NCWS FCP +), whereas all IBS/FD patients proved negative (P = 0.0001). FCP using a cut-off value > 41 µg/g showed a 58.6% sensitivity and a 98.0% specificity (AUC 0.755, 95% C.I. 0.702–0.837) in distinguishing NCWS from IBS/FD patients. Of the 63 NCWS FCP+, 65.1% had negative FCP values after ≥ 6 months of WFD, with a significant reduction in FCP values (*P* < 0.0001). All NCWS FCP- subjects still preserved negative FCP values after ≥ 6 months of WFD. Our study showed that FCP can be a useful but supplementary diagnostic marker for differentiating between NCWS and IBS/FD. Strict WFD adherence reduced FCP values, normalizing them in 65.1% of NCWS FCP + subjects. These data suggest the existence of two NCWS subgroups: NCWS FCP + characterized by a probable predominantly inflammatory/immunologic pattern and NCWS FCP− featuring non-immuno-mediated etiopathogenetic mechanisms. (Registration number NCT01762579).

**Supplementary Information:**

The online version contains supplementary material available at 10.1007/s11739-024-03595-7.

## Introduction

Non-celiac gluten sensitivity (NCGS) has emerged as a gluten-related disorder [1], characterized by gastrointestinal symptoms [overlapping with irritable bowel syndrome (IBS) and/or functional dyspepsia (FD)] and extraintestinal manifestations (e.g., headache, neurological impairment, and asthenia) following gluten/wheat ingestion in the absence of celiac disease (CD) or wheat allergy (WA) [2]. To date a non-invasive marker has not been identified; thus, a double-blind, placebo-controlled challenge (DBPCC) with gluten has been suggested as the gold standard for NCGS diagnosis [3]. Although this test is scientifically adequate, only about 30% of suspected patients respond. This result could be due to the inherent heterogeneity and complexity of NCGS [4], so additional markers are needed to better identify these patients.

In addition, conflicting data on the pathophysiology of the disease have been reported, and other components of wheat, different from gluten, have been proposed as potential triggers. Therefore, the term NCGS has been replaced with a more appropriate expression: non-celiac wheat sensitivity (NCWS) [5].

Among these wheat components, amylase-trypsin inhibitors (ATIs) [6] could have a pathophysiological role, modifying intestinal permeability and activating innate immunity through interaction with Toll-Like Receptor-4 (TLR-4) expressed on the monocytes, macrophages, and dendritic cells of the intestinal mucosa [7, 8]. Moreover, ATIs might be responsible for an “indirect” activation of the adaptive response, extending from the gut to other organs, resulting in NCWS extraintestinal manifestations [9]. Other factors have been proposed as possible triggers of intestinal permeability impairment and the activation of inflammatory mechanisms: (1) change in gut microbiota composition [10, 11]; (2) potential “toxic” effect of gluten/gliadin[12, 13]; and (3) non-IgE-mediated allergic response to gluten/wheat proteins [14–16].

Supporting the inflammatory hypothesis, some studies have proved an increase in cytokine levels related to innate immunity both in the serum and in intestinal mucosa specimens, especially in the rectum [7, 17–19].

Nevertheless, this inflammatory hypothesis has been rejected by some authors, who have sometimes also denied the existence of NCWS [20, 21], underlining the role of FODMAPs as possible mechanical triggers of NCWS clinical manifestations [10, 21, 22].

Fecal calprotectin (FCP) is a non-invasive and non-expensive marker of intestinal inflammation, whose role in both the diagnosis and monitoring of inflammatory bowel disease (IBD) has received international consensus [23]. However, many other conditions (i.e., collagenous colitis, neoplastic disease, diverticulitis, CD, and food allergies) have been associated with increased FCP levels, as intestinal inflammation is a feature common to all of them [24–26]. The degree of increased FCP values differs, depending on the cause, and is relevant in the differential diagnosis between patients with functional gastrointestinal symptoms and IBD [24, 25, 27].

Assuming the NCWS inflammatory pathogenetic hypothesis to be reliable, this study aimed to verify whether FCP could be used to identify/confirm the underlying inflammatory status in NCWS patients, and whether this biomarker could be useful to differentiate NCWS from IBS/FD. Our final aim was to verify whether adopting a strict wheat-free diet (WFD) can considerably modify FCP values in NCWS patients, thus indirectly proving a reduction in the inflammatory status.

## Methods

We conducted both a retrospective and prospective multicenter study. To identify eligible subjects, we reviewed the charts of patients with NCWS diagnosed by DPBCC with wheat between January 2007 and June 2022 in 3 third-level centers for gluten-related disorders: Unit of Internal Medicine, V. Cervello Hospital of Palermo, Italy; Department of Internal Medicine, University Hospital of Palermo, Italy; Department of Internal Medicine, Hospital of Sciacca, Italy. As a control group, we selected age- and sex-matched patients with IBS/FD, diagnosed according to the Rome III and IV criteria [28, 29] in the same period and in the same centers, and with a clinical history unrelated to food allergies/intolerances.

### Inclusion and exclusion criteria for patient recruitment

To select both the NCWS and IBS/FD patients to be recruited in this study, the same inclusion and exclusion criteria already used and validated in several other research papers conducted in this field by our study group were applied [2, 15, 18, 26, 30–32]. For details, see Online Source 1 and 2.

The application of these criteria (including a follow-up longer than 12 months, with at least 2 outpatient visits during this period) excluded all patients in whom an intestinal and/or extraintestinal disease might have caused the reported symptoms and produced a consequent increase in FCP values (e.g., CD, infectious or inflammatory bowel disease, including microscopic colitis, ulcerative colitis and Crohn’s disease, neoplastic disease, diverticulitis, segmental colitis associated with diverticulosis, food allergies, etc.). For this purpose, all patients underwent abdominal and intestinal ultrasound examination, and when clinically required on the basis of the signs and symptoms reported by patients, other imaging (computerized tomography and/or magnetic resonance) and/or endoscopic examinations (including esophagogastroduodenoscopy, rectoscopy, and/or colonoscopy with biopsies) were performed.

### Outcomes

#### Primary outcome: prevalence of FCP positivity and definition of its role as a diagnostic biomarker in NCWS patients

The retrospective phase of our study aimed to identify the prevalence of FCP positivity in an NCWS population with a certain diagnosis and contextually define its role as a diagnostic biomarker to differentiate NCWS from IBS/FD subjects. To this purpose, the medical records of patients with NCWS and IBS/FD not related to gluten/wheat intolerance, which had been previously validated in retrospective studies [30–32], were analyzed for their demographic, clinical, genetic, histological, and laboratory features (details provided in Online Source 1).

All the data were collected in an electronic database and analyzed to estimate the prevalence of these features, as well as the mean/median values of blood and fecal parameters both in the NCWS and IBS/FD subjects. The population was then stratified according to the positivity/negativity of FCP, to identify any putative features differentiating the subgroups. Finally, we attempted to define the potential diagnostic power of FCP values in differentiating NCWS from IBS/FD subjects. For the Method details see Online Source 1.

#### Secondary outcome: effects of a strict WFD on FCP values in NCWS subjects

In the prospective phase of our study, all the NCWS patients recruited in the retrospective phase were recontacted, to assess their adherence to the WFD. Compliance to the diet was evaluated by a validated questionnaire, based on a modified version of the Pavia/Biagi score questionnaire where 0 = no adherence to the WFD; 1–2 = poor adherence; and 3–4 = excellent adherence [33–35].

All the patients were again invited to follow a strict WFD, especially those who had been noncompliant or reported poor adherence to the WFD. All those who accepted were recalled every month to check adherence (using the same score) and to ensure motivational reinforcement. All the NCWS patients with an ascertained ≥ 6-month period of strict WFD were asked to repeat the FCP assay.

#### Determination of FCP values

For the collection of the fecal samples, the patients used a collection scoop with which a small amount of feces was obtained and then placed in a sterile container, without any preservatives. The sample was stored in a refrigerator and delivered to the laboratory within 24 h after collection. Analyses of fecal samples were all performed at the University Hospital of Palermo to reduce variability (the intra-assay coefficient of variation was 5.2%, the inter-assay coefficient was 7.1%). FCP levels were assessed by a commercially available quantitative enzyme immunosorbent test (Calprest, Eurospital, Trieste, Italy). A small amount of feces (median 100 mg, range 40–120 mg) was initially diluted at a weight–volume ratio of 1:50; the extraction solution was then added to the sample. After 30 s of vortexing, the sample was homogenized for about 25 min and finally centrifuged for 20 min at room temperature. The dilution buffer was subsequently added to 0.5 mL of the supernatant to obtain a 1:50 dilution. According to the manufacturer’s instructions, this product was placed in duplicate in the wells of the plate. Two processing cycles, each including an incubation of about 45 min and three washing operations, were performed to obtain 100 µL of enzyme substrate, which were added to each well. Finally, after a further 45-min incubation cycle at room temperature, an experienced biologist recorded the optical density (absorbance) at a wavelength of 405 nm. The results were calculated using the manufacturer’s instructions and the FCP values of each sample were expressed in µg/g, using > 50 µg/g as the positivity cut-off value.

### Statistical analysis

Data were expressed as mean ± standard deviation (SD) when the distribution was Gaussian and Student’s *t*-test was used to evaluate differences between the groups. Comparisons between more than 2 groups were performed with ANOVA, followed by a post hoc analysis using the Bonferroni test. Otherwise, data are expressed as median (range) and interquartile range (IQR) and analyzed with the Mann–Whitney U or Wilcoxon signed rank test. The χ^2^ test and Fisher’s exact test were used to compare frequency values across population groups.

The receiver operating characteristic (ROC) curve was constructed by calculating the sensitivity and specificity of individual FCP values, and the corresponding area under the curve (AUC) was calculated to evaluate the diagnostic accuracy of the tests in differentiating NCWS vs IBS/FD subjects [36].

The SPSS Statistics version 27.0, and MedCalc version 22.0 software were used for the statistical analysis.

This study was registered on ClinicalTrials.gov (registration number NCT01762579) and approved by the Ethics Committee of the University Hospital of Palermo (Record n.10/2019).

## Results

**Fig. 1:**
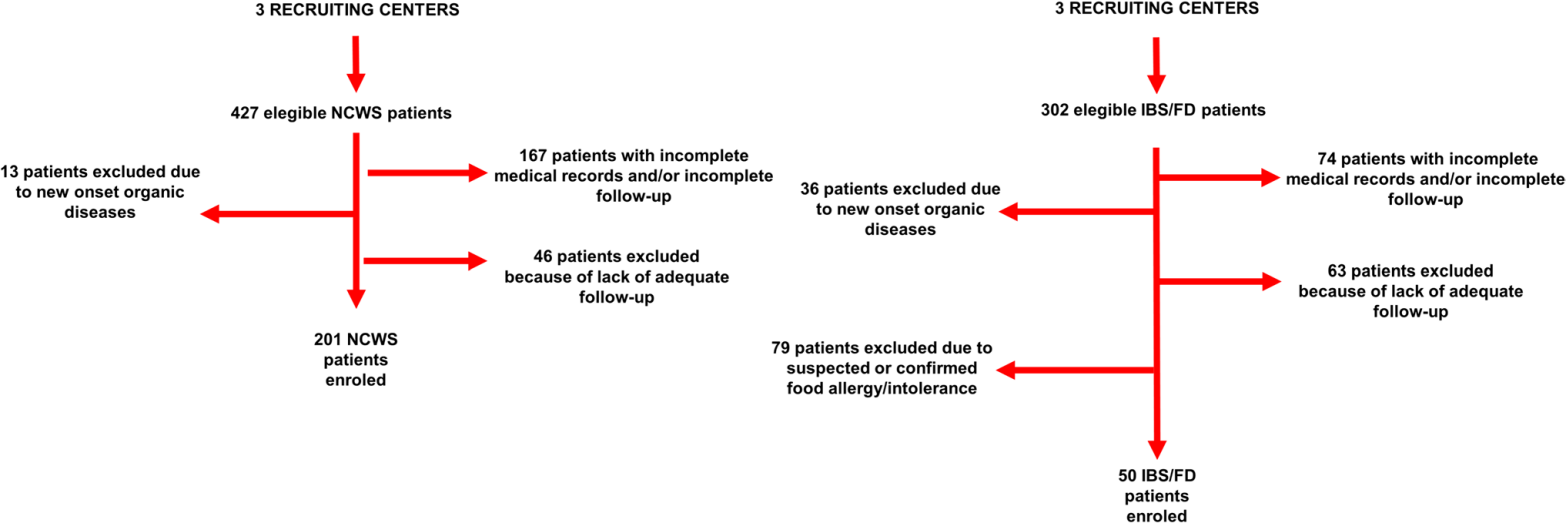
Flowchart of the retrospective part of this study. *IBS/FD* irritable bowel syndrome/functional dyspepsia, *NCWS* non-celiac wheat sensitivity

The clinical records of 427 NCWS and 302 IBS/FD patients were reviewed. After the application of the inclusion/exclusion criteria, 201 NCWS and 50 IBS/FD patients were found eligible and then recruited (Fig. 1)*.*

### Primary outcome: prevalence of FCP positivity and definition of its role as a diagnostic biomarker in NCWS patients

**Fig. 2:**
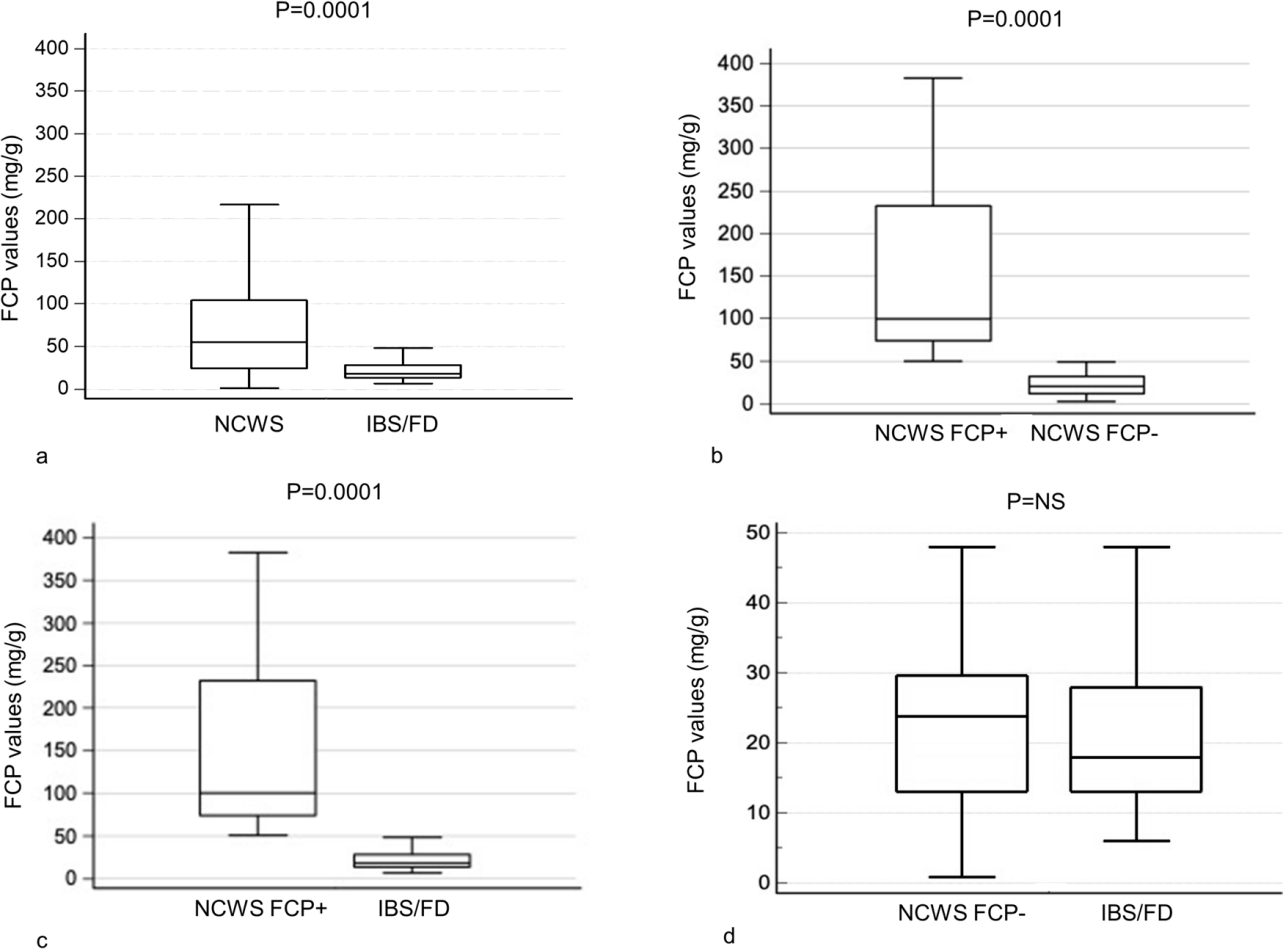
Pre-diet FCP values in NCWS compared to IBS/FD (**a**); NCWS FCP + compared to NCWS FCP− (**b**); NCWS FCP + compared to IBS/FD (**c**); NCWS FCP− compared to IBS/FD (**d**). Values expressed as median and IQR. *FCP *fecal calprotectin, *IBS/FD* irritable bowel syndrome/functional dyspepsia, *IQR *interquartile range, *NCWS* non-celiac wheat sensitivity, *NCWS FCP +* NCWS with positive FCP values, *NCWS FCP− *NCWS with negative FCP values

Before diagnosis and therefore prior to the WFD, 31.3% (*n* = 63) of the 201 NCWS patients had FCP values above normal limits (NCWS FCP+), whereas all the IBS/FD patients proved negative (*P* = 0.0001). Moreover, absolute FCP values were significantly higher in the NCWS than in the IBS/FD patients (*P* = 0.0001); more specifically, this significance was determined by the subgroup of NCWS FCP + patients (*P* = 0.0001 when compared with both NCWS patients with negative FCP (NCWS FCP−) and with IBS/FD patients), whereas no difference in FCP mean values was found between NCWS FCP− and IBS/FD patients (Table 1; Fig. 2).

**Table 1:** FCP of patients with NCWS and IBS/FD recruited in this study before and after WFD

	Total NCWS *N* = 201 A	NCWS FCP + *N* = 63 B	NCWS FCP− *N* = 138 C	IBS/FD *N* = 50 D	*P*
FCP on wheat-containing diet (*n*, %)					
Negative Positive	138 (68.7) 63 (31.3)*	– –	– –	50 (100.0) 0 (0.0)	A vs D 0.0001
FCP values on wheat-containing diet (mg/g)					
Median (min–max) IQR	59** (3–559) 25.0–103.1	112*** (54–559) 79.1–236.2	24^§^ (3–47) 13.0–32.2	18 (6–48) 13.1–28.3	A vs D 0.0001 B vs C 0.0001 B vs D 0.0001
FCP on WFD (*n*, %)	*N* = 95	*N* = 63	*N* = 32		
Negative Positive	73 (76.8) 22* (16.8)	41 (65.1) 22 (34.9)	32 (100.0) 0 (0.0)	– –	B vs C < 0.0001
FCP values on WFD (mg/g)	*N* = 95	*N* = 63	*N* = 32		
Median (min–max) IQR	30** (2–240) 16.0–54.0	103*** (9–240) 54.1–167.0	21^§^ (3–35) 12.5–30.0	– –	B vs C 0.0001

*IBS/FD* irritable bowel syndrome/functional dyspepsia, *FCP* fecal calprotectin, *IQR* interquartile range, *NCWS* non-celiac wheat sensitivity, *NCWS FCP +* NCWS with positive FCP values, *NCWS FCP− *NCWS with negative FCP values, *NS* not significant, *WFD* wheat-free diet

^*^P = 0.02

^**^Z = 5.6; *P* = 0.0001

^***^Z = 3.29; *P* < 0.0001

^§^*Z* = 4.6; *P* = 0.001

Tables 2 and 3 show the demographic, clinical, genetic, and histological features of the NCWS patients (both of the whole group and then stratified into subgroups according to FCP positivity) compared with those of IBS/FD subjects. As reported in “Materials and Methods,” duodenal and rectal/colon biopsy sampling was performed only in patients whose clinical, laboratory, and imaging features required further investigation to exclude organic diseases; thus, 125 (62.2%) and 76 (37.8%) NCWS subjects underwent duodenal and rectal/colon histology examination, respectively. Likewise, 5 (10.0%) and 7 (14.0%) IBS/FD patients underwent the same histological analyses.

**Table 2:** Demographic and clinical characteristics of patients with NCWS and IBS/FD enrolled in this study

	Total NCWS *N* = 201 A	NCWS FCP + *N* = 63 B	NCWS FCP− *N* = 138 C	IBS/FD *N* = 50 D	*P*
Sex (*n*, %)					
Females Males	175 (87.1) 26 (12.9)	51 (81.0) 12 (19.0)	124 (89.9) 14 (10.1)	48 (96.0) 2 (4.0)	NS NS
Age at diagnosis (years), (mean ± SD)	37.4 ± 12.1	38.2 ± 18.0	37.0 ± 11.8	41.6 ± 13.6	NS
Age at onset (years), (mean ± SD)	28.0 ± 12.7	28 ± 14	28.1 ± 12.1	31 ± 15	NS
Diagnostic delay (months), median (IQR)	60 (24–120)	60 (24–156)	60 (24–120)	105.8 (12–162)	NS
BMI (kg/m^2^), (mean ± SD)	23.5 ± 5	21 ± 4	24.3 ± 5.1	29 ± 5.3	NS
IBS-like symptoms, (*n*, %)					
None Diarrhea Constipation Mixed bowel movements	17 (8.5) 106 (52.7) 23 (11.4) 55 (27.4)	4 (6.3) 37 (58.7) 6 (9.5) 16 (25.4)	13 (9.4) 69 (59.0) 17 (12.3) 39 (28.3)	1 (2.0) 20 (40.0) 11 (22.0) 18 (36.0)	NS NS NS NS
Dyspepsia, (*n*, %)	116 (57.7)	32 (50.8)	84 (60.9)	27 (54.0)	NS
Weight loss, (*n*, %)	60 (29.9)	23 (36.5)	37 (26.8)	7 (14.0)	A vs D 0.043
Extraintestinal Symptoms, (*n*, %)	145 (72.1)	40 (63.5)	105 (76.1)	25 (50.0)	A vs D 0.004
Menstrual cycle alterations^a^, (*n*, %)	57/173 (32.9)^a^	18/57 (31.5)^a^	39/116 (33.6)^a^	6/45 (13.3)^a^	A vs D 0.0001
Autoimmune diseases, (*n*, %)	48 (23.9)	18 (28.6)	30 (21.7)	2 (4.0)	A vs D 0.016 B vs D 0.0007 C vs D 0.042
Hashimoto's thyroiditis, (*n*, %)	28 (13.9)	8 (12.7)	20 (14.5)	1 (2.0)	A vs D 0.018 B vs D 0.037 C vs D 0.016
SRMI (*n*, %)	133 (66.2)	39 (61.9)	94 (68.1)	17 (34.0)	A vs D 0.0001
Atopy (*n*, %)	58 (28.9)	14 (22.2)	44 (31.9)	13 (26.0)	NS

*BMI* Body Mass Index, *IBS/FD* irritable bowel syndrome/functional dyspepsia, *IQR* interquartile range, *FCP *fecal calprotectin, *NCWS* non-celiac wheat sensitivity, *NCWS FCP +*  NCWS with positive FCP values, *NCWS CP− *NCWS with negative FCP values, *NS* not significant, *SD* standard deviation, *SRMI* self-reported milk intolerance

^a^Not including menopausal and/or hysterectomy patients

**Table 3:** Genetic and histologic characteristics of patients with NCWS and IBS/FD recruited in this study

	Total NCWS *N* = 201 A	NCWS FCP + *N* = 63 B	NCWS FCP- *N* = 138 C	IBS/FD *N* = 50 D	*P*
HLA DQ2/DQ8, (*n*, %)					
Negative	91 (45.3)	28 (44.4)	63 (45.7)	21 (42.0)	NS
Positive	110 (54.7)	35 (55.6)	75 (54.3)	29 (58.0)	NS
Marsh–Oberhuber classification, (*n*, %)					
0 1	59/125 (47.2) 66/125 (52.8)	14/39 (35.9) 25/39 (64.1)	45/86 (52.3) 41/86 (47.7)	5/5 (100.0) 0 (0.0)	A vs D 0.0001 B vs D 0.0001 C vs D 0.0001
Eosinophils, (n, %)					
Duodenum Colon Rectum	31/125 (24.8) 24/54 (44.4) 32/76 (42.1)	15/39 (38.5) 10/22 (45.5) 13/30 (43.3)	16/86 (18.6) 14/32 (43.8) 19/46 (41.3)	0/5 (0.0) 0/7 (0.0) 0/2 (0.0)	A vs D 0.005 A vs D 0.0001 A vs D 0.0001

*FCP* fecal calprotectin, *HLA* human leukocyte antigens, *IBS/FD *irritable bowel syndrome/functional dyspepsia, *NCWS *non-celiac wheat sensitivity, *NCWS FCP +* NCWS with positive FCP values, *NCWS FCP− *NCWS with negative FCP values, *NS* not significant

The NCWS group was more likely than the IBS/FD patients to suffer from weight loss (*P* = 0.043), extraintestinal symptoms (*P* = 0.004), menstrual cycle alterations (*P* = 0.0001), autoimmune disorders (*P* = 0.016), SRMI (*P* = 0.0001), and to have a Marsh 1 (*P* = 0.0001) at duodenal histology and eosinophil infiltration in the intestinal mucosa (*P* = 0.005, *P* = 0.0001 and *P* = 0.0001 at duodenum, colon, and rectum biopsies, respectively). In the subgroup analysis, no differences were found in the NCWS FCP + or NCWS FCP- patients, whether between the two subgroups or when compared with IBS/FD patients, except for a higher frequency in both the subgroups than in the IBS/FD patients of Marsh 1 (*P* = 0.0001, for both) and autoimmune disorders (*P* = 0.0007 for NCWS FCP + and *P* = 0.042 for NCWS FCP-). In addition, the possibility of a correlation between FCP values and the subtype of IBS-like symptoms reported by patients in all the groups was investigated, but no statistically significant differences could be found (see Online Source 2).

The blood chemistry data analysis showed that compared to IBS/FD patients the NCWS patients (overall and both NCWS FCP + and NCWS FCP-) had a higher frequency of elevated TSH (*P* = 0.0018, *P* = 0.037 and *P* = 0.016, respectively) and of ANA positivity (*P* = 0.0001 for all) (see Online Source 2).

**Fig. 3:**
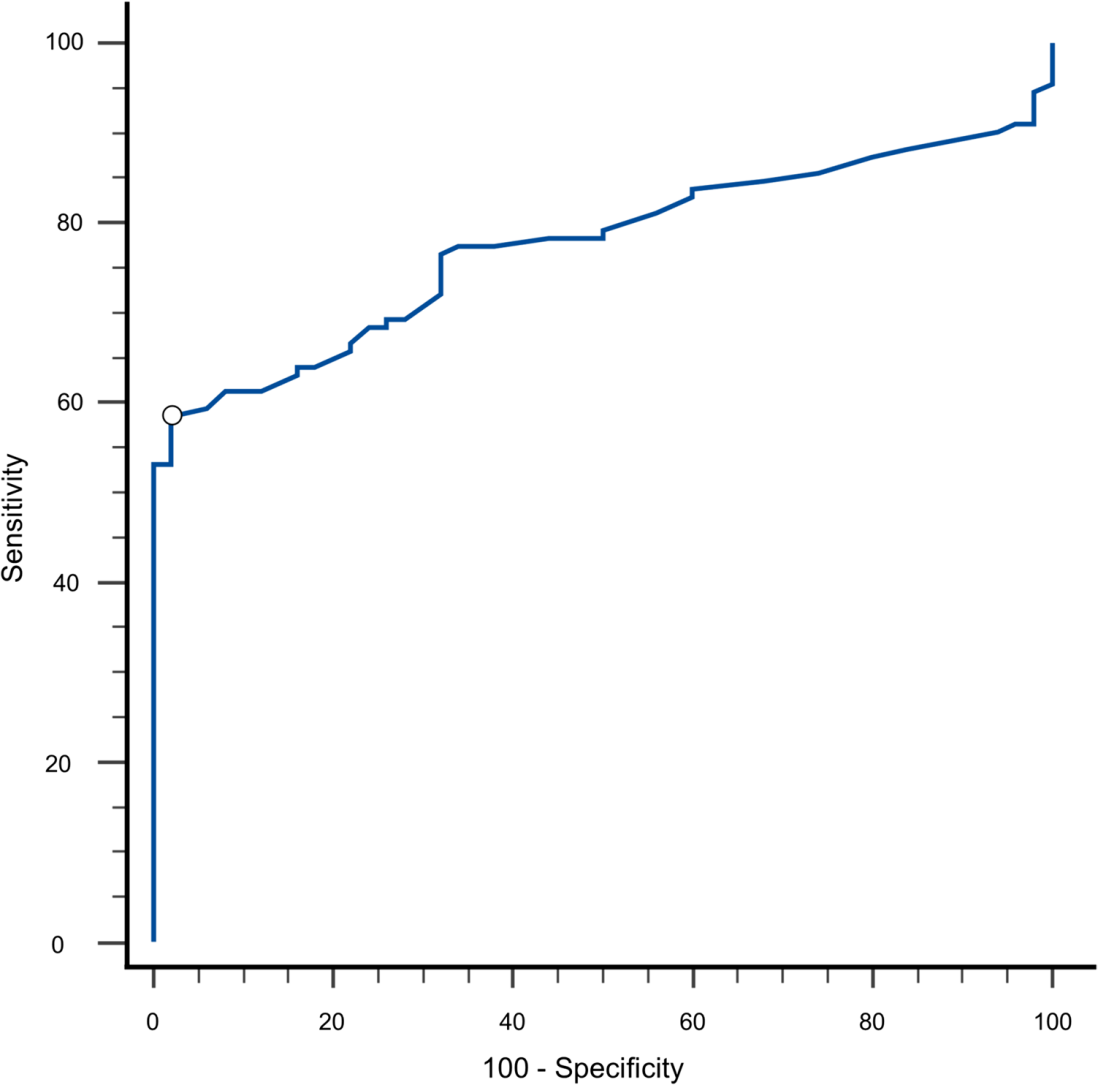
ROC curve analysis of FCP values. *FCP* fecal calprotectin, *ROC* receiver operating characteristic

Finally, the diagnostic performance of FCP values in differentiating NCWS from IBS/FD patients was tested. Figure 3 shows the ROC curve analysis for FCP in the NCWS and IBS/FD patients, with an AUC of 0.755 (confidence interval 95%, 0.702–0.837). Using an FCP cut-off value > 41 µg/g, this analysis showed that it is possible to distinguish NCWS from IBS/FD patients with a 58.6% sensitivity and 98.0% specificity, and a positive likelihood ratio (PLR) of 29.3.

### Secondary outcome: effects of a strict WFD on FCP values in NCWS subjects

The NCWS patients (*n* = 201) included in the retrospective phase of the study were recalled, to be enrolled in the prospective phase. Preliminary adherence to WFD was assessed by physicians with experience in gluten-related diseases; more than half of the patients (50.8%) reported following a strict WFD (i.e., modified Biagi score 3–4), without significant differences between NCWS FCP + and FCP− (Online Source 2). All patients, regardless of the results of the adherence score, were invited to follow a strict WFD for at least 6 months; thereafter, all subjects for whom a ≥ 6-month period of strict WFD could be ascertained were requested to repeat the FCP assay.

All the NCWS FCP + patients (29 with adherence scores 0–2; 34 with adherence scores 3–4) were enrolled in the prospective study and repeated the FCP assay as requested. Among the 70 NCWS FCP− patients with adherence scores 0–2, 15 agreed to follow a strict WFD for ≥ 6 months and subsequently undergo a FCP assay. Of the 68 NCWS FCP- with adherence scores 3–4, 17 were enrolled in this study and repeated the FCP assay. Consequently, taken together all the NCWS FCP + patients were reassessed after ≥ 6 months on a strict WFD, while only 32 pre-WFD NCWS FCP- patients participated in the prospective phase of the study (Online Source 3).

**Fig. 4:**
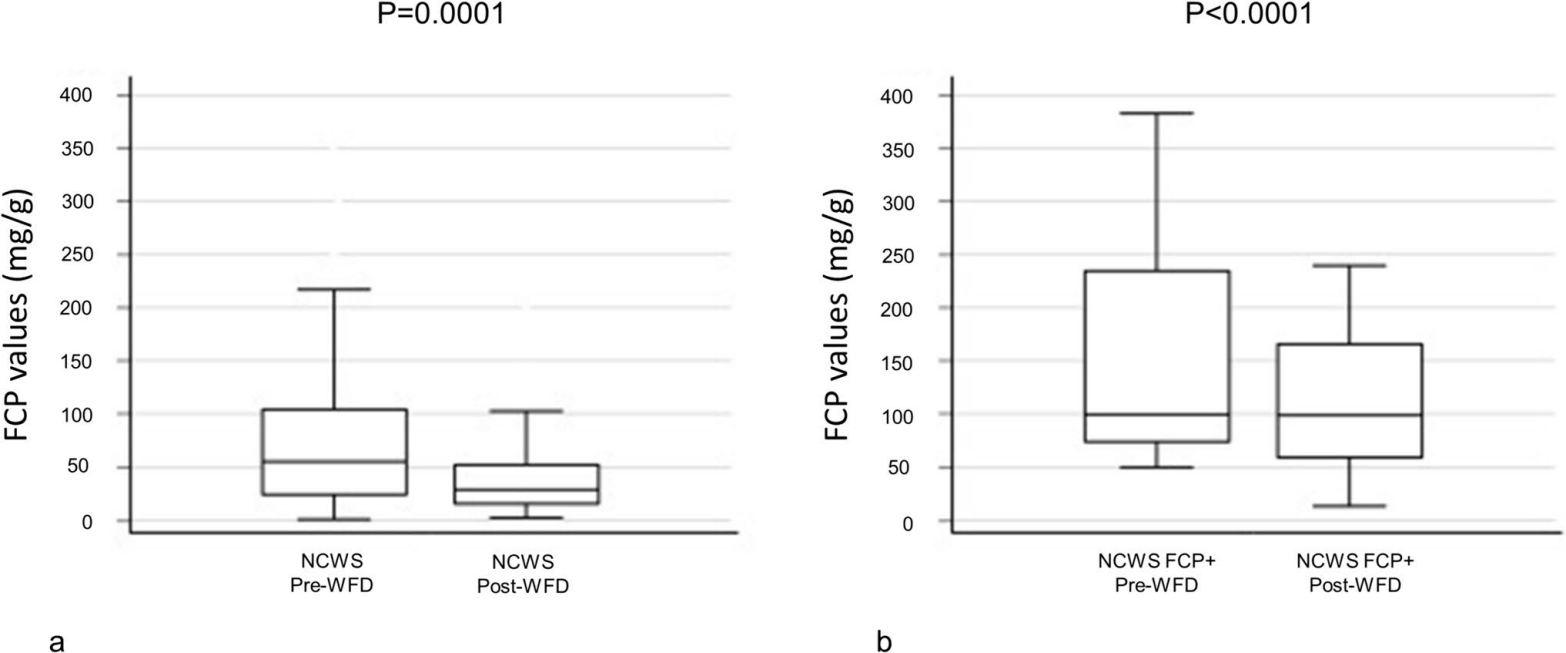
Pre- and post-WFD FCP values in: total NCWS (**a**); NCWS FCP + (**b**) Values expressed as median and IQR. *FCP* Fecal Calprotectin, *IQR* Interquartile Range, *NCWS* non-celiac wheat sensitivity, *NCWS FCP +* NCWS with positive FCP values, *WFD* wheat-free diet

The prevalence of high FCP values in the NCWS patients after ≥ 6 months of "strict" WFD was 16.8%, significantly lower than the 31.3% reported before the start of the WFD (*P* = 0.02). Moreover, FCP values showed a significant reduction between pre- and post-WFD [median (IQR): 59 (25.0–103.1) mg/g vs 30 (16.0–54.0) mg/g, *Z* = 5.6, *P* = 0.0001] (Table 1 and Fig. 4, Panel a).

Of the 63 NCWS FCP + subjects before diagnosis, 65.1% had negative FCP values after ≥ 6 months of WFD, with a significant reduction in values between pre- and post-WFD [median (IQR): 112 (79.1–236.2) mg/g vs 103 (54.1–167.0) mg/g, *Z* = 3.29, *P* < 0.0001] (Table 1 and Fig. 4, Panel b). All the NCWS FCP- subjects before diagnosis still had negative FCP values after ≥ 6 months of WFD, but with a significant reduction in values between pre- and post-WFD [median (IQR): 24 (13.0–32.2) mg/g vs 21 (12.5–30.0) mg/g, *Z* = 4.6, *P* = 0.001] (Table 1). Finally, comparing the reduction in FCP after ≥ 6 months of WFD in the two NCWS subgroups, a significantly greater reduction was shown in NCWS FCP + patients (*P* = 0.0001) (Table 1).

## Discussion

To date, the etiopathogenetic bases of NCWS still remain one of the most obscure sides of the disease. Among these, several studies have stressed the importance of intestinal permeability alterations, resulting in an activation of the immune system [10, 11] (both innate[14, 19] and adaptive [18]) by gluten [12, 13] and/or other wheat components (e.g., ATIs) [6, 8, 9, 14–16], as proved by rectal histology [18, 19], whereas contrasting data are reported in duodenal findings [37].

In consideration of these results, it is possible to assume that the colon/rectum might be the main site of both the alterations in intestinal permeability and inflammation; thus, a possible inflammatory marker of disease could be FCP, a calcium-binding protein, which is primarily found in neutrophils and macrophages and whose presence in stools is probably due to the migration of myeloid cells into the intestinal mucosa [24]. This marker is known to have a high negative predictive value in differentiating IBD from functional gut diseases, such as IBS [38]. However, the presence of low-grade inflammation in IBS may still cause a slight increase in FCP values, so FCP has a poor positive predictive value for IBD diagnosis [38].

Considering, therefore, the role of FCP in distinguishing between patients with inflammatory and functional disorders of the gut and the probable scenario of chronic intestinal inflammation in patients with NCWS, we investigated whether FCP could be used to identify/confirm this underlying inflammatory status in patients with a certain diagnosis of NCWS. In our study, FCP values higher than the normal limit were found in 31.3% of NCWS patients on a wheat-containing diet, in contrast to 0.0% among IBS/FD patients (*P* = 0.0001). Stratifying our study population according to the original diagnosis and FCP values, we identified three subgroups of patients: NCWS FCP+, NCWS FCP−, and IBS/FD. FCP values in the NCWS FCP + subjects were higher than in the NCWS FCP− and IBS/FD patients (*P* = 0.0001, for both). Of note, the FCP values in the NCWS FCP− subjects were virtually identical to those with IBS/FD, thus suggesting an underlying inflammatory process in NCWS FCP + subjects and a non-inflammatory one in NCWS FCP− and IBS/FD, which might share the same pathogenetic mechanisms. When analyzing the demographic, clinical, genetical, histological, and laboratory features, we observed no differences between the 3 subgroups except for the statistically significant higher prevalence of autoimmune diseases and Marsh 1 histology in the whole NCWS group and its subgroups, when compared to the IBS/FD group.

Considering both the frequency of FCP positivity in the NCWS study group and the widely variable values obtained [59 (25.0–103.1) mg/g], it could be hypothesized that the NCWS population examined was heterogeneous and composed of at least two subgroups of patients. The first subgroup was characterized by symptoms related to a prevalent immunological/inflammatory mechanism (i.e., NCWS FCP+); instead, in the second one the reported symptoms were probably caused by a non-immune mediated/non-inflammatory mechanism (i.e., NCWS FCP−). The latter could be similar to a food intolerance, like the one induced by FODMAPs in patients with IBS/FD (gut distention, with consequent induced abdominal pain via intestinal mechanoreceptor activation) [21, 22].

The hypothesis of a prevalent inflammatory/immunological involvement in patients with NCWS FCP + is also supported by both the higher frequency of Marsh 1 in duodenal biopsies compared to both NCWS FCP- (not statistically significant) and IBS/FD (*P* = 0.0001), although the limited number of duodenal biopsies might have influenced the results. It is reasonable to suppose that a higher number of biopsies, obtained both at the duodenum and rectum, could confirm a significant difference not only between NCWS and IBS/FD but also between NCWS FCP + and NCWS FCP− patients.

Starting from these data, which made us suspect the existence of an inflammatory/immunological substrate in a subgroup of NCWS patients, we evaluated whether FCP values would change after 6 months of strict WFD. Adoption of a strict WFD seemed to “turn off” the inflammatory substrate in the intestinal mucosa, as demonstrated not only by the lower frequency of FCP positivity (31.3% pre-WFD vs 16.8% post-WFD, *P* = 0.02) but also by the reduction in FCP values (*P* = 0.0001).

Among the NCWS FCP + patients, 65.1% had negative FCP values after 6 months of WFD and a concomitant sharp reduction in absolute values (*P* < 0.0001). Similarly, also in the NCWS FCP− subjects a significant reduction in FCP values (*P* < 0.0001) after WFD could be seen, although this “anti-inflammatory effect” of WFD was more evident in the NCWS FCP + than in the NCWS FCP− patients (*P* = 0.0001).

As for the diagnostic performance of FCP in the NCWS patients, the ROC curve analysis showed an AUC of 0.755 and a 58.6% sensitivity and 98.0% specificity for FCP values > 41 µg/g. Thus, based on these values the PLR was 29.3, so that assuming a pre-test probability of 7% (mean prevalence value of NCWS in subjects with IBS-like symptoms) [2, 3, 39, 40], the post-test probability of NCWS was 69%. This analysis, although carried out on a limited number of patients, suggests that FCP could be a useful additional tool in the evaluation of patients with an IBS/FD-like clinical presentation and with a suspected NCWS.

Other authors have tried to identify putative diagnostic biomarkers for NCWS. Uhde M. et al. showed that AGA IgG levels were significantly higher in NCWS subjects compared to healthy controls (*P* < 0.0001), but no difference was found when compared to CD patients. The same authors analyzed this marker in association with other markers of systemic inflammation and intestinal permeability, proving the existence of a systemic immune activation related to intestinal permeability imbalance in NCWS. They did not identify a single potential biomarker, and thus suggested that a panel of markers might be helpful [11]. Nevertheless, other studies seem to exclude the role of AGA IgG as a diagnostic marker in NCWS [41]. Consequently, isolated AGA IgG positivity in a context of intestinal/extraintestinal symptoms is usually considered just a potential clue toward NCWS [3]. Similarly, another group, who proved a 70.9% sensitivity and an 83.1% specificity of serum zonulin levels in discriminating between NCWS and diarrhea-prevalent IBS patients, proposed a diagnostic score based on gender, zonulin serum levels, and the severity of abdominal pain and distension [42].

NCWS diagnosis is still based on the Salerno criteria [3]; thus, the identification of a non-invasive marker with a specificity of 98% means that if a patient with IBS/FD-like symptoms and who self-perceives a gluten/wheat sensitivity, without other organic diseases, shows FCP values > 41 µg/g, a diagnosis of NCWS could be made without performing a DBPCC. On the contrary, FCP values < 41 mg/g should increase the likelihood of a functional non-inflammatory disorder.

This finding is in agreement with the international literature data: a meta-analysis showed that for FCP values lower than 40 µg/g, there was a 1% chance of having IBD, a 14.9% chance of IBS, and an 84.1% chance of being a healthy control [38].

The obvious message of our work, therefore, is that it is certainly not conceivable to rely exclusively on FCP values to diagnose NCWS. However, in a context in which the patient's willingness to undergo a DBPCC with wheat is often extremely low, a finding of FCP values > 41 µg/g can help differentiate between at least a subgroup of NCWS patients (i.e., the NCWS FCP + , “inflammatory” subgroup) and IBS/FD subjects, thus allowing patients to avoid the food challenge. Thus, in our opinion, FCP assays should be added to the panel of markers used to evaluate NCWS patients. Unfortunately, we were not able to analyze the markers proposed by both Udhe et al. [11] and Barbaro et al. [42], so we cannot propose a diagnostic algorithm including FCP; a study with this specific purpose is required.

The abnormal FCP values of NCWS subjects identified in our analysis might shed new light on the NCWS etiopathogenetic mechanisms, based on the possible presence of an inflammatory/immunological component.

In this context, some authors have reported that gliadin and its fragments possess a neutrophil chemoattractant activity, both through the production of IL-8 in CD patients, and through the activation of the Met-Leu-Phe receptor-1 pathway in mouse models [43]. Thus, as a marker of neutrophil activation [44], the high levels of FCP found before diagnosis in our NCWS population, and their reduction after a 6-month period of strict WFD, seem to strengthen the idea of a chronic inflammatory status induced by wheat exposure.

Our study has several limitations. First, since it is a partly retrospective study, a selection bias may have occurred. Second, the rather small number of recruited patients in both the NCWS and IBS/FD groups may have resulted in a beta-type error and prevented some of the parameters examined from reaching statistical significance. The very retrospective nature of our study did not allow us to analyze other markers of intestinal and systemic inflammation in order to clarify if the hypothesis of the two different subsets of NCWS patients (i.e., inflammatory vs non-inflammatory subgroup) could be confirmed. In addition, the limited number of pre-WFD biopsies obtained, especially in the control population, may have considerably affected the results. In the prospective part of the study, the limitations are related to the low number of recruited patients with pre-WFD negative FCP values, as well as to the total absence of a biopsy follow-up after the 6-month "strict" WFD. Another shortcoming of our analysis is linked to the absence of a correlation between the FCP values and a scale to evaluate the severity of patient symptoms (e.g., the irritable bowel severity scoring system). Finally, our study lacks an external validation group, one possibly not recruited in a third-level center for the diagnosis and treatment of gluten-related disorders. Due to all these limitations, the obtained results must be considered as preliminary and not potentially extendable to the entire NCWS population. Thus, a prospective multicenter study with a much higher number of patients is required to confirm our data.

The strengths of the study, on the other hand, are that all the recruited NCWS patients were diagnosed after a DBPCC with wheat, and that FCP values were evaluated only after a congruous and lengthy period of strict WFD. Nevertheless, it is noteworthy that the DBPCC with wheat is a diagnostic tool which, due to its low sensitivity [3, 4], tends to hyper-select the patients included in this study, who are certainly affected by NCWS, but which may not represent the entire NCWS population, potentially excluding some subjects with pathophysiological mechanisms different from those discussed in our paper. Therefore, we remark that data from this study are potentially valid only for a subgroup of patients with NCWS (i.e., DBPCC with wheat positive NCWS patients).

## Conclusions

Our study showed that FCP, with a specificity of 98% at a cut-off value of 41 µg/g, can be a useful supplementary diagnostic marker in the differential diagnosis between NCWS and IBS/FD patients, but only after all possible organic causes that could lead to the symptoms reported by the patients have been ruled out.

Moreover, based on the evidence of the different behaviors of FCP both pre-WFD and after 6 months of strict adherence to WFD, but also of some histopathological findings, it seems possible to identify at least two distinct subgroups of NCWS patients. The first is characterized by a probable prevailing inflammatory/immunologic pattern and with pathologic pre-WFD FCP values, which decline considerably after WFD; the other features non-immuno-mediated etiopathogenetic mechanisms on a chronic inflammatory substrate, in which pre-WFD FCP values are within normal limits and decline less markedly after WFD. Thus, our findings reinforce the idea that NCWS is a protean condition in which different subgroups of patients characterized by different pathophysiological mechanisms (some of which are probably shared with IBS/FD, i.e., NCWS FCP- patients) coexist, united by a single trigger: the ingestion of wheat.

### Supplementary Information

Below is the link to the electronic supplementary material.

Supplementary file1 (DOCX 51 KB)

Supplementary file2 (DOCX 27 KB)

## Data Availability

The data presented in this study are available on request from the corresponding author. The data are not publicly available due to restrictions of patient privacy.
