# Supplementary material for: Fecal calprotectin levels in patients with non-celiac wheat sensitivity: a proof of concept
Source: Intern Emerg Med. 2024 Apr 12;19(5):1255–66. doi: 10.1007/s11739-024-03595-7 (PMC11364563; doi:10.1007/s11739-024-03595-7)
Supplement: Supplementary file 1 — Supplementary file1 (DOCX 51 KB) [file 11739_2024_3595_MOESM1_ESM.docx]

**Fecal calprotectin levels in patients with Non-Celiac Wheat Sensitivity: A proof of concept**

**Journal name: Internal and Emergency Medicine**

**Aurelio Seidita (1,2), Alessandra Giuliano (1), Maurizio Soresi (3), Marta Chiavetta (1), Emilio Nardi (4), Giuseppe Mogavero (5), Giulio Giannone (6), Antonio Carroccio (1), Pasquale Mansueto (3)**

1. Unit of Internal Medicine, “V. Cervello” Hospital, Ospedali Riuniti “Villa Sofia-Cervello”, Palermo, Italy, and Department of Health Promotion Sciences, Maternal and Infant Care, Internal Medicine and Medical Specialties (PROMISE), University of Palermo, Palermo, Italy

2. Institute for Biomedical Research and Innovation (IRIB), National Research Council (CNR), Palermo, Italy

3. Unit of Internal Medicine, Department of Health Promotion Sciences, Maternal and Infant Care, Internal Medicine and Medical Specialties (PROMISE), University of Palermo, Palermo, Italy

Specialties (PROMISE), University of Palermo, Palermo, Italy

4. Unit of Internal Medicine II, Department of Health Promotion Sciences, Maternal and Infant Care, Internal Medicine and Medical Specialties (PROMISE), University of Palermo, Palermo, Italy

Specialties (PROMISE), University of Palermo, Palermo, Italy

5. Unit of Gastroenterology, “V. Cervello” Hospital, Ospedali Riuniti “Villa Sofia-Cervello”, Palermo, Italy

6. Pathology Unit, Department of Health Promotion Sciences, Maternal and Infant Care, Internal Medicine and Medical Specialties (PROMISE), University of Palermo, Palermo, Italy

**Address for correspondence**: Antonio Carroccio, Internal Medicine, Via Ciaculli 207, 90124, Palermo, Italy. Mail address: [antonio.carroccio@unipa.it](mailto:antonio.carroccio@unipa.it)

Inclusion criteria for Non-Celiac Wheat Sensitivity (NCWS) patients

- Age ≥18 and ≤65 years;

- Patients with: 1) both intestinal and extra-intestinal wheat-dependent symptoms; 2) negative anti-deamidated gliadin protein (DGP) immunoglobulins (Ig) class A (IgA) and IgG, anti-tissue transglutaminase (tTG) IgA and IgG, and endomysial antibodies (EmA) IgA; 3) absence of duodenal villous atrophy in biopsy specimens of all the patients with DQ2 and/or DQ8 haplotypes, when they had had a minimum intake of 100 g of pasta and/or bread for at least 30 days; 4) exclusion of wheat allergy (WA) (negative skin prick-test and/or serum specific IgE for wheat, gluten and gliadin); 5) resolution/reduction of symptoms on a strict standard elimination diet (i.e. oligoantigenic diet, without wheat, cow’s milk, and other foods reported by the patients themselves as causing symptoms) followed for at least 4 weeks, and then the reappearance of the same symptoms after a DBPCC with wheat (for the elimination diet and challenge methods see below);

- Complete clinical records

- Follow-up longer than 12 months, with at least 2 outpatient visits during this period.

Exclusion criteria for NCWS patients

- Age ≤17 e ≥66 years

- Self-exclusion of gluten/wheat from the diet and refusal to reintroduce it for diagnostic purposes before entering the study

- EmA positivity in the culture medium of duodenal mucosa samples (EmA-biopsy), even in the presence of a normal villus/crypt ratio in the mucosa

- Pregnancy or breastfeeding

- Alcohol and/or drug abuse

- Treatment with corticosteroids and/or nonsteroidal anti-inflammatory drugs in the 2 weeks prior to performing duodenal biopsies

- Diagnosis of inflammatory bowel disease (IBD) or other organic pathologies of the digestive system [including infectious bowel disease, microscopic colitis, diverticulitis, segmental colitis associated with diverticulosis), neoplastic disease (both intestinal and extraintestinal)], disease of the nervous system, major psychiatric disorders, infective pathologies, immunodeficiency and impairments limiting physical activity

- Incomplete clinical records or period of follow-up shorter than 12 months after diagnosis.

Inclusion criteria for Irritable Bowel Syndrome/Functional Dyspepsia (IBS/FD) patients

- Age ≥18 e ≤65 years;

- Subjects with IBS-like symptoms and/or FD, according to the Roma III [1] or Roma IV [2] criteria, who did not refer intestinal or extraintestinal symptoms related to wheat and whose symptoms were not reduced or did not improve on a gluten-/wheat-free diet,

- Complete clinical records

- Period of follow-up longer than 12 months after diagnosis, with at least 2 outpatient visits during this period.

Exclusion criteria for IBS/FD patients

- Age ≤17 e ≥66 years

- Pregnancy or breastfeeding

- Alcohol and/or drug abuse

- Treatment with corticosteroids and/or nonsteroidal anti-inflammatory drugs in the 2 weeks prior to duodenal biopsies and prior to fecal sampling for fecal calprotectin (FCP) assay

- Diagnosis of IBD or other organic pathologies of the digestive system [including CD, infectious bowel disease, microscopic colitis, diverticulitis, segmental colitis associated with diverticulosis), neoplastic disease (both intestinal and extraintestinal)], disease of the nervous system, major psychiatric disorders, infective pathologies, immunodeficiency, and impairments limiting physical activity

- Suspected or confirmed food allergy/intolerance

- Incomplete clinical records or period of follow-up shorter than 12 months after diagnosis

**Standard elimination diet and Double-Blind Placebo-Controlled Challenge (DBPCC)**

To confirm the diagnosis of Non-Celiac Wheat Sensitivity after other organic conditions had been excluded, all patients who self-reported gluten/wheat sensitivity started a standard elimination diet, without wheat, cow’s milk, eggs, tomato and chocolate. Patients self-reporting multiple food hypersensitivity were also asked to avoid ingestion and/or contact with other food(s) causing symptoms. Food diaries were kept during the elimination diet period to assess dietary intake and adherence to the diet. After 4 weeks of elimination diet, a DBPCC was performed, with the reintroduction of a single food at a time. Patients were randomized to receive either the “active food” or the placebo, according to a computer-generated order determined by an observer not involved in the study.

The DBPCC was performed with sachets of flour coded A or B containing wheat flour or rice flour, respectively. Sachets A or B were given for 2 consecutive weeks, then after 1 week of washout patients received the other sachets for another 2 weeks (cross-over design). If needed, the washout period was extended for a maximum of another 2 weeks until the symptoms induced by the previous challenge had completely resolved before the next challenge was started. Wheat challenges were performed by administering a daily dose of 80g of flour, which was dissolved and cooked by the patients themselves. Wheat sachets contained 6.5g of gluten, and an estimated 0.3g of amylase trypsin inhibitors (ATIs), as determined by bioassay [3].

The codes of the sachets were broken only at the end of the study and the investigators did not know their contents during the study period. Challenges for other foods in patients with suspected multiple food hypersensitivity were performed in an open fashion. During the challenge period, the severity of intestinal symptoms was recorded: patients completed a 100mm visual analog scale (VAS, with 0 representing no symptoms, and 10 intolerable symptoms), which assessed overall symptoms as well as the specific symptoms they each reported. The challenges were stopped when clinical reactions occurred for at least two consecutive days (increase in VAS score >30, both for irritable bowel syndrome-like symptoms - onset of abdominal discomfort or pain, associated with a change in stool frequency and/or stool appearance - and for extraintestinal symptoms). Challenges were considered positive if the same symptoms which had been initially present reappeared after their disappearance on the elimination diet and if the VAS score was >30 when compared to any eventual increase recorded during the placebo administration.

**Demographic, clinical, genetical, histological and laboratory features analyzed in the retrospective phase of the study**

The following demographic, clinical, genetical, histological and laboratory features were collected and analyzed in the retrospective phase of the study: sex, age at diagnosis (years), age at symptom onset (years), diagnostic delay (months), body mass index (BMI, Kg/m^2^), IBS-like symptoms (diarrhea, constipation, mixed bowel movements), dyspepsia, weight loss, extraintestinal symptoms, menstrual cycle alterations, autoimmune disorders, self-reported milk intolerance (SRMI), atopy (allergic rhinitis and/or asthma and/or atopic dermatitis), HLA-DQ2/DQ8 haplotype, duodenal histology (according to  the Marsh-Oberhuber classification) [4,5], presence of eosinophils in duodenal, colon and rectal histology, and values of thyroid-stimulating hormone (TSH), anti-nuclear antibodies (ANA), extractable nuclear antigen antibodies (ENA), anti-gastric parietal cell antibodies (APCA) and FCP.

**HLA DQ2/DQ8 determination**

DQ2 and DQ8 haplotypes were identified using a commercial kit (Xeligen, Eurospital, Trieste, Italy) according to the manufacturer’s instructions, by Real Time Polymerase Chain Reaction on DNA extracted from peripheral blood samples.

**Autoimmune Disease prevalence**

Patients were considered as suffering from autoimmune diseases when diagnosis was confirmed by an experienced rheumatologist (or endocrinologist in the case of Hashimoto’s thyroiditis) according to the international guidelines for each specific disease. For example, for the diagnosis of primary Sjögren's Syndrome an experienced rheumatologist confirmed diagnosis according to the 2016 American College of Rheumatology/European League Against Rheumatism Classification Criteria for Primary Sjögren's Syndrome [6].

**Systemic nickel allergy syndrome (SNAS) and atopy prevalence**

Patients were considered as having SNAS when they proved positive to a nickel patch test and reported symptoms improvement after at least one month on a low nickel diet [7]. Only a few patients underwent oral patch test with nickel sulphate.

Patients were considered as suffering from atopy when one of the following conditions was confirmed by an experienced allergologist, based on clinical manifestations, skin prick tests, patch tests, and/or total/specific Immunoglobulin (Ig) E evaluation: allergic rhinitis and/or asthma, allergic rhinitis and/or atopic dermatitis.

**Duodenal histology analysis**

Oriented formalin-fixed paraffin-embedded biopsies were used to prepare serial sections (4mm) of duodenal mucosa. Intestinal biopsy specimens were classified according to the Marsh-Oberhuber classification [8,9] and stained by an immune-enzymatic method for the labeling of CD3+ IELs. CD3+ IEL density was calculated as the percentage of enterocytes.

The number of eosinophils per high-power field (×40) was also assessed; the upper limit of the reference interval in our laboratory was 60 EOS/10 high-power fields.

**Colon and rectum histology analysis**

Biopsy specimens were obtained from subjects who underwent colonoscopy and/or proctoscopy even in the case of a macroscopically healthy mucosa. The number of eosinophils was assessed as previously described by our group [10].

**Thyroid-stimulating hormone (TSH) and autoantibody analysis**

TSH levels were assayed on serum samples with a commercial kit (Thyroid Stimulating Hormone Human ELISA Kit, Thermo Fisher Scientific, Waltham, Massachusetts, United States), according to the manufacturer’s instructions.

Anti-nuclear antibodies (ANA), extractable nuclear antigen antibodies (ENA) and anti-gastric parietal cell antibodies (APCA) were tested on serum samples collected during the visits and stored at -20 °C, using the methods we previously described [11].

**References**

1. Drossman DA. The functional gastrointestinal disorders and the Rome III process. Gastroenterology [Internet]. 2006 [cited 2023 Nov 12];130(5):1377–90. Available from: https://pubmed.ncbi.nlm.nih.gov/16678553/
2. 39. Lacy BE, Mearin F, Chang L, Chey WD, Lembo AJ, Simren M, et al. Bowel Disorders. Gastroenterology [Internet]. 2016 May 1 [cited 2023 Nov 12];150(6):1393-1407.e5. Available from: <https://pubmed.ncbi.nlm.nih.gov/27144627/>
3. Marsh MN. Gluten, major histocompatibility complex, and the small intestine. A molecular and immunobiologic approach to the spectrum of gluten sensitivity ('celiac sprue’) - PubMed [Internet]. [cited 2023 Nov 12]. Available from: https://pubmed.ncbi.nlm.nih.gov/1727768/
4. Oberhuber G, Granditsch G, Vogelsang H. The histopathology of coeliac disease: time for a standardized report scheme for pathologists. Eur J Gastroenterol Hepatol [Internet]. 1999 [cited 2023 Nov 12];11(10):1185–94. Available from: https://pubmed.ncbi.nlm.nih.gov/10524652/
5. Zevallos VF, Raker VK, Maxeiner J, Scholtes P, Steinbrink K, Schuppan D (2019) Dietary wheat amylase trypsin inhibitors exacerbate murine allergic airway inflammation. Eur J Nutr 58(4):1507-1514. doi: 10.1007/s00394-018-1681-6.
6. Shiboski CH, Shiboski SC, Seror R, et al (2017) International Sjögren's Syndrome Criteria Working Group. 2016 American College of Rheumatology/European League Against Rheumatism Classification Criteria for Primary Sjögren's Syndrome: A Consensus and Data-Driven Methodology Involving Three International Patient Cohorts. Arthritis Rheumatol 69(1):35-45. doi: 10.1002/art.39859.
7. Ricciardi L, Arena A, Arena E, et al (2014) Systemic nickel allergy syndrome: epidemiological data from four Italian allergy units. Int J Immunopathol Pharmacol 27(1):131-6. doi: 10.1177/039463201402700118.
8. Marsh MN (1992) Gluten, major histocompatibility complex, and the small intestine. A molecular and immunobiologic approach to the spectrum of gluten sensitivity ('celiac sprue'). Gastroenterology 102(1):330-54.
9. Oberhuber G, Granditsch G, Vogelsang H (1999) The histopathology of coeliac disease: time for a standardized report scheme for pathologists. Eur J Gastroenterol Hepatol 11(10):1185-94. doi: 10.1097/00042737-199910000-00019.
10. Carroccio A, Giannone G, Mansueto P, et al (2019) Duodenal and Rectal Mucosa Inflammation in Patients With Non-celiac Wheat Sensitivity. Clin Gastroenterol Hepatol 17(4):682-690.e3. doi: 10.1016/j.cgh.2018.08.043.
11. Mansueto P, Soresi M, Candore G, et al (2021) Autoimmunity Features in Patients With Non-Celiac Wheat Sensitivity. Am J Gastroenterol 1;116(5):1015-1023. doi: 10.14309/ajg.0000000000000919.
