# Supplementary material for: Fecal calprotectin levels in patients with non-celiac wheat sensitivity: a proof of concept
Source: Intern Emerg Med. 2024 Apr 12;19(5):1255–66. doi: 10.1007/s11739-024-03595-7 (PMC11364563; doi:10.1007/s11739-024-03595-7)
Supplement: Supplementary file 2 — Supplementary file2 (DOCX 27 KB) [file 11739_2024_3595_MOESM2_ESM.docx]

**Fecal calprotectin levels in patients with Non-Celiac Wheat Sensitivity: A proof of concept**

**Journal name: Internal and Emergency Medicine**

**Aurelio Seidita (1,2), Alessandra Giuliano (1), Maurizio Soresi (3), Marta Chiavetta (1), Emilio Nardi (4), Giuseppe Mogavero (5), Giulio Giannone (6), Antonio Carroccio (1), Pasquale Mansueto (3)**

1. Unit of Internal Medicine, “V. Cervello” Hospital, Ospedali Riuniti “Villa Sofia-Cervello”, Palermo, Italy, and Department of Health Promotion Sciences, Maternal and Infant Care, Internal Medicine and Medical Specialties (PROMISE), University of Palermo, Palermo, Italy

2. Institute for Biomedical Research and Innovation (IRIB), National Research Council (CNR), Palermo, Italy

3. Unit of Internal Medicine, Department of Health Promotion Sciences, Maternal and Infant Care, Internal Medicine and Medical Specialties (PROMISE), University of Palermo, Palermo, Italy

Specialties (PROMISE), University of Palermo, Palermo, Italy

4. Unit of Internal Medicine II, Department of Health Promotion Sciences, Maternal and Infant Care, Internal Medicine and Medical Specialties (PROMISE), University of Palermo, Palermo, Italy

Specialties (PROMISE), University of Palermo, Palermo, Italy

5. Unit of Gastroenterology, “V. Cervello” Hospital, Ospedali Riuniti “Villa Sofia-Cervello”, Palermo, Italy

6. Pathology Unit, Department of Health Promotion Sciences, Maternal and Infant Care, Internal Medicine and Medical Specialties (PROMISE), University of Palermo, Palermo, Italy

**Address for correspondence**: Antonio Carroccio, Internal Medicine, Via Ciaculli 207, 90124, Palermo, Italy. Mail address: [antonio.carroccio@unipa.it](mailto:antonio.carroccio@unipa.it)

**Supplementary Table 1. FCP values (mg/g) of NCWS and IBS/FD patients enrolled in the study according to the reported IBS/FD-like symptoms.**

|  | **None** | **Diarrhea** | **Constipation** | **Mixed bowel movements** | ***P*** |
| --- | --- | --- | --- | --- | --- |
| Total NCWS  N=201 A  Median (min-max)  IQR | N=17  53 (8-264)  11.4-233.6 | N=106  57 (3-559)  25.7-138.5 | N=23  55 (3-126)  20.0-103.0 | N=55  55 (3-383)  15.5-122.1 | NS |
| NCWS FCP+  N=63 B  Median (min-max)  IQR | N=4  106 (61-264)  116.75-256.25 | N=37  119 (54-559)  75.5-247.25 | N=6  102 (55-383)  85.25-243.5) | N=16  107 (55-383)  81.5-276.5 | NS |
| NCWS FCP-  N=138 C  Median (min-max)  IQR | N=13  11 (6-47)  8.2-32.0 | N=69  24 (3-45)  13-32.25 | N=17  20 (3-42)  11.5-31.5 | N=39  16 (3-42)  7.0-33.5 | NS |
| IBS/FD  N=50 D  Median (min-max)  IQR | N=1 | N=20  20 (9-36)  13.5-27.5 | N=11  19 (6-41)  13.1-33.3 | N=18  17 (11-48)  13.8-30.2 | NS |

*FCP=Fecal Calprotectin; IBS/FD=Irritable Bowel Syndrome/Functional Dyspepsia; IQR=Interquartile Range; NCWS=Non-Celiac Wheat Sensitivity; NCWS FCP+=NCWS with positive FCP values; NCWS FCP-=NCWS with negative FCP values; NS=Not Significant.*

**Supplementary Table 2. Blood chemistry features of NCWS and IBS/FD patients enrolled in the study.**

|  | **Total NCWS**  **N=201**  **A** | **NCWS FCP+**  **N=63**  **B** | **NCWS FCP-**  **N=138**  **C** | **IBS/FD**  **N=50**  **D** | ***P*** |
| --- | --- | --- | --- | --- | --- |
| TSH (μIU/ml) (n, %)  Normal range    Above normal range (n, %)    Below normal range (n, %) | 172 (85.6)  28 (13.9)  1 (0.5) | 54 (85.7)  8 (12.7)  1 (1.6) | 117 (84.8)  20 (14.5)  1 (0.7) | 48 (96.0)  1 (2.0)  1 (2.0) | A vs D 0.045  B vs D 0.049  C vs D 0.038  A vs D 0.018  B vs D 0.037  C vs D 0.016  NS |
| ANA positive (n, %) | 115 (57.2) | 37 (58.7) | 78 (56.5) | 6 (12.0) | A vs D 0.0001  B vs D 0.0001  C vs D 0.0001 |
| ANA titers (n, %)  1:80  1:160  1:320  1:640 | 70 (60.9)  19 (16.5)  16 (13.9)  10 (8.7) | 23 (62.2)  9 (24.3)  3 (8.1)  2 (5.4) | 47 (60.3)  10 (12.8)  14 (17.9)  7 (9.0) | 3 (50.0)  2 (33.3)  1 (16.7)  0 (0.0) | NS  NS  NS  NS |
| ENA positive (n, %) | 20 (9.9) | 6 (9.5) | 14 (10.1) | 2 (4.0) | NS |
| APCA positive (n, %) | 31 (15.4) | 13 (20.6) | 18 (13.0) | 1 (2.0) | A vs D 0.0001  B vs D 0.0001  C vs D 0.0001 |

*ANA=Anti-Nuclear Antibodies; APCA=Anti-Parietal Cells Antibodies; FCP=Fecal Calprotectin; ENA=Extractable Nuclear antigen Antibodies; IBS/FD=Irritable Bowel Syndrome/Functional Dyspepsia; NCWS=Non-Celiac Wheat Sensitivity; NCWS FCP+=NCWS with positive FCP values; NCWS FCP-=NCWS with negative FCP values; NS=Not Significant; TSH=Thyroid Stimulating Hormone.*

**Supplementary Table 3. Adherence to wheat-free diet of NCWS patients enrolled in the study before the start of the prospective phase, according to the modified version of Biagi/Pavia Score [1-3]**

|  | **Total NCWS**  **N=201**  **A** | **NCWS FCP+**  **N=63**  **B** | **NCWS FCP-**  **N=138**  **C** | ***P*** |
| --- | --- | --- | --- | --- |
| Modified Adherence score  0  1  2  3  4 | 75 (37.3)  13 (6.5)  11 (5.4)  13 (6.5)  89 (44.3) | 22 (34.9)  4 (6.4)  3 (4.8)  5 (7.9)  29 (46.0) | 53 (38.4)  9 (6.5)  8 (5.8)  8 (5.8)  60 (43.5) | NS  NS  NS  NS  NS |

*FCP=Fecal Calprotectin; NCWS=Non-Celiac Wheat Sensitivity; NCWS FCP+=NCWS with positive FCP values;*

*NCWS FCP-=NCWS with negative FCP values; NS=Not Significant*

1. Seidita A, Mansueto P, Giuliano A, et al (2022) Potential tolerability of ancient grains in non-celiac wheat sensitivity patients: A preliminary evaluation. Front Med (Lausanne) 28;9:995019. doi: 10.3389/fmed.2022.995019.
2. Mansueto P, Seidita A, Soresi M, et al (2023). Anemia in non-celiac wheat sensitivity: Prevalence and associated clinical and laboratory features. Dig Liver Dis Jun;55(6):735-742. doi: 10.1016/j.dld.2022.11.022.
3. Biagi F, Bianchi PI, Marchese A, et al. (2021) A score that verifies adherence to a gluten-free diet: a cross-sectional, multicentre validation in real clinical life. Br J Nutr 108: 1884-8. doi: 10.1017/S0007114511007367.
